# Sociodemographic Factors Associated with Physical Functioning in Elderly Males and Females from Serbia: Population-Based Modeling Study

**DOI:** 10.3390/healthcare13091028

**Published:** 2025-04-30

**Authors:** Milena Kostadinovic, Dejan Nikolic, Ardak Nurbakyt, Dinara Sukenova, Bojana Matejic, Ivana Sotirovic, Natasa Mujovic, Filip Milanovic, Ljubica Nikcevic, Milena Santric-Milicevic

**Affiliations:** 1 University Clinical Center of Serbia, 11000 Belgrade, Serbia; milena8250@gmail.com (M.K.); natasa.mujovic@med.bg.ac.rs (N.M.); 2 Faculty of Medicine, University of Belgrade, 11000 Belgrade, Serbia; ljubicanikcevic@yahoo.com; 3 Department of Physical Medicine and Rehabilitation, University Children’s Hospital, 11000 Belgrade, Serbia; 4 Department of Public Health, Asfendiyarov Kazakh National Medical University, Almaty 050012, Kazakhstan; a.nurbakyt@kaznmu.kz (A.N.); sukenova.d@kaznmu.kz (D.S.); 5 School of Public Health, Faculty of Medicine, University of Belgrade, 11000 Belgrade, Serbia; bojana.matejic@med.bg.ac.rs (B.M.); ivana.sotirovic@med.bg.ac.rs (I.S.); milena.santric-milicevic@med.bg.ac.rs (M.S.-M.); 6 Department of Pediatric Surgery, University Children’s Hospital, 11000 Belgrade, Serbia; filipmilanovic333@gmail.com; 7 Special Hospital for Cerebrovascular Disease “Saint Sava”, 11000 Belgrade, Serbia

**Keywords:** sociodemographic factors, physical functioning, gender, elderly

## Abstract

**Background and aim:** During the past few decades, the aging population has increased. With aging, there is an increase in functional limitations. The aim of this study was to analyze sociodemographic factors associated with physical functioning impairment in elderly males and females. **Methods**: This population-based modeling study based on a data from a third national study of health of Serbian inhabitants from 2013 in Serbia included 3540 elderly participants 65 years of age and above from Serbia. Physical functioning for both genders was categorized as follows: PF1—walking half a kilometer on level ground without the assistance of any mobility aids and PF2—walking up or down 12 steps. Modeling of physical functioning for both genders was categorized as follows: Model 1: inability to perform PF1, Model 2: some/a lot of difficulty in performing PF1, Model 3: inability to perform PF2, and Model 4: some/a lot of difficulty in performing PF2. Further variables were evaluated: age, education level, marital status, body mass index (BMI), wealth index, and place of residence. Logistic regression was performed to identify the variables that are factors associated with PF1 and PF2 in elderly males and females. **Results**: Statistically significant factors were as follows: age (Model 1 (male OR: 2.591; female OR: 4.708); Model 2 (male OR: 1.791; female OR: 2.354); Model 3 (male OR: 2.386; female OR: 4.985); Model 4 (male OR: 1.883; female OR: 2.772)); BMI (Model 2 (female OR: 1.348); Model 4 (female OR: 1.329)), marital status (Model 2 (female OR: 0.713); Model 4 (male OR: 0.688)); education level (Model 1 (male OR: 0.626; female OR: 0.537); Model 2 (male OR: 0.811; female OR: 0.653); Model 3 (male OR: 0.697; female OR: 0.494); Model 4 (male OR: 0.784; female OR: 0.639)); wealth index (Model 2 (male OR: 0.823; female OR: 0.740); Model 3 (male OR: 0.724); Model 4 (male OR: 0.787; female OR: 0.731)); and place of residence (Model 1 (female OR: 1.704); Model 3 (female OR: 1.575)). **Conclusions**: Increased age, being single, a lower education level, and a lower wealth index were factors associated with functional disability in the elderly of both genders, while an increased BMI and living in another place than a city were factors associated with functional disability in elderly females. Specific social strategies bearing in mind possible gender differences should be created and implemented in order to optimize the physical functioning, mobility, and participation of the elderly.

## 1. Introduction

During the past few decades, the aging population has increased. The possible factors that can contribute to this are increasing longevity, declining fertility [1], and improvement in quality of life [2]. According to the World Health Organization (WHO), in 2024, life expectancy at birth was 73.3 years, which was an 8.4-year increase since 1995 [3]. It is stated that one in six people globally by 2030 will be 60 years of age or over [4]. Moreover, it is projected that by the late 2070s, the population of individuals aged 65 and older will surpass that of children under 18 years on a global scale [5]. This demographic shift and increasing longevity underscore the critical need to adapt social structures to enhance self-care capabilities. The demographic issues faced by the Republic of Serbia involve a declining population, lower fertility rates, and aging demographics, leading to a one-third rise in the proportion of individuals aged 65 and older over a ten-year period, increasing from 17.4% in 2011 to 22.0% in 2022, according to the report by the Statistical Office of the Republic of Serbia [6,7]. Applying the biopsychosocial lenses to the development of necessary health and care support requires an understanding of the main factors contributing to individuals who are aging with some disabilities and those who are transitioning into disability [8]. These factors remain less discussed, concerning the conventional perceptions of gender roles in society.

There is a wide range of abilities in performing activities of daily living (ADLs), instrumental ADLs, and responses to disability in the elderly [8]. It is stated that disability is a dynamic process that can be influenced by numerous factors [9]. In a national health survey analyzed from Serbia, sociodemographic predictors associated with physical functioning in the elderly were found to be gender, age, marital status, and education levels [10]. In a study from Canada on community-dwelling older adults, significantly higher scores on the physical activity scale for the elderly were noticed in males, those that were not living alone or in seniors housing, older adults that were married or in common-law, and those with a higher level of education and higher income [11]. Furthermore, the study that was performed on Greek elderly participants demonstrated that physical functioning limitations were more profound in females and the older [12]. Socioeconomic status was found to have a significant impact on disability in older adults, with the dominant role of economic conditions mainly in health behavior and life security in China [13]. Moreover, both childhood and adult socioeconomic status were shown to relate to functional disability in older adults according to a study from China based on the data from the 2013 wave of the Cina Health and Retirement Longitudinal Study (CHARLS) [14]. Additionally, a study based on the Survey on Disability, Personal Autonomy, and Dependency Situations from the Spanish National Statistics Institute demonstrated that low income and educational levels are associated with difficulties in performing ADLs in non-institutionalized men and women 65 years and older [15].

As people age, there is an accumulation of health problems, and performing ADLs are getting challenged [16]. In a study on the Greek elderly population, it was noted that stroke and cerebrovascular diseases, hip and femoral fractures, arthritis or rheumatism, Parkinsons, and cataracts were strongly associated with functional impairment [12]. It is pointed out that around 30% of community-dwelling adults 65 years of age and above fall each year, with an increase in such proportion to 50% in those over 75 years of age [17]. A cross-sectional survey of individuals from 18 to 94 years revealed several risk factors distinguishing individuals with a physical disability who reported falling versus those who did not, including age, gender, mobility level, imbalance, number of comorbid conditions, and diagnosis duration [18]. The consequences of falls in the elderly are numerous, including the fear of falling, losing confidence, deconditioning, functional decline, and, in some cases, institutionalization and ultimately, death [17]. Therefore, early recognition of possible factors that can be associated with physical and functional decline in aging individuals and implementation of preventive measures that would lead to the reduction in fall rates in older men and women will improve overall health, society participation, and overall quality of life in elderly individuals.

Additionally, it was reported that muscle function phenotypes are highly heritable, where certain genetic loci were described to be associated with physical performance phenotypes [19]. The alfa-actinin-3 (ACTN3) R577X polymorphism was shown to have an influence on the response of quadricep muscle power to strength training in older adults [20]. Moreover, in a study on the Japanese population, it was noticed that the ACTN3 R577X genotype is associated with muscle function in the lower extremities [21].

A better understanding of the factors that can be potentially associated with physical functioning impairments in elderly males and females, as well as their differences, could lead to the proposal of gender-specific, optimal preventive measures and interventional strategies in social and healthcare sectors impacting adequate functioning and influencing the overall health status and quality of life of this population. Given the abovementioned, we have hypothesized that in elderly males and females, different sociodemographic factors could be associated with different disability degrees regarding physical functioning. Therefore, the aim of this study was to analyze sociodemographic factors associated with physical functioning impairment in elderly males and females.

## 2. Material and Methods

### 2.1. Study Participants

We performed population-based modeling study as a secondary and retrospective analysis from Institute of Public Health (IPH) electronic database in Serbia on 3540 individuals aged ≥65 years. Ministry of Health of the Republic of Serbia evaluated the health of Serbian inhabitants within the third national study, “Istraživanje zdravlja stanovništva Srbije u 2013” [22], and conducted in accordance with the European Health Interview Survey wave 2 (EHIS wave 2) methodology [22].

Institutional Review Board of the Faculty of Medicine, the University of Belgrade in Belgrade, Serbia, approved this study (No: 29/III-8).

### 2.2. Study Criteria Selection

National representative probability sample was created by a census of individuals, households, and apartments from the year 2011. Population data from 2011 in Serbia were considered in order to perform stratification of the representative sample, with two variables (region and settlement type) included for initial stratums creation, where four statistical regions were identified as the main strata: Vojvodina, Belgrade, Sumadija with Western Serbia, and Southern with Eastern Serbia, that were, based on cities and other areas, further divided into eight strata. A two-step sampling method was utilized for creating sample distribution on the national level. Probability proportional sampling (670 census areas) was performed first, followed by household selection by a simple random sample without replacement regarding census areas (10 households and 3 additional spare households). The representative sample included 6500 households, with 3540 (24.2%) elderly older persons ≥ 65 years [22].

The inclusion criteria were private household residents in the Republic of Serbia, while exclusion criteria were collective household residents, residents of geriatric institutions, and those who refused to participate in this study.

### 2.3. Study Parameters

Further parameters were analyzed according to gender separately for males and females: age of individuals, where elderly were divided into three groups: Group 1—between 65 and 74 years; Group 2—between 75 and 84 years; and Group 3—above 85 years [10,23]. Regarding education level, the elderly were classified into three groups: elementary school (≤8 years of education), high school (between 9 and 12 years of education), and university (>12 years of education) [10]. Considering marital status, study participants were categorized into single and married. Body mass index (BMI) analysis was performed by the body height and weight measures that were taken and calculated as kg/m^2^. According to the WHO classification, study participants were classified into four BMI categories: underweight (<18.50), normal weight (18.50–24.99), overweight (≥25.00), and obese (≥30.00) [24]. Regarding the place of residence, elderly participants were grouped into the ones living in the city or the ones living in another place.

### 2.4. Wealth Index

The Demographic and Health Survey Wealth Index, or Wealth Index estimation, was previously described, including variables related to the property excluding income [25,26]. The Serbian household wealth index is ranked into five socioeconomic categories (the richest, rich, middle class, poor, and the poorest) [27]. The wealth index categorization for the purpose of this study was modified into three categories: lower (poor and poorest), middle (middle class), and upper (rich and richest) [10,27].

### 2.5. Difficulty in Walking Modalities

Physical functioning (PF) capacity in this study was assessed by analysis of two categories of walking difficulties for males and females in order to model four different difficulty levels of PF. These categories were considered as factors that are capable of assessing the capacity for effort in walking.

The first category of walking difficulty (PF1) evaluated the ability to walk half a km on level ground without the use of any aid. For this purpose, the formulated question was, “Do you have difficulty walking half a km on level ground that would be [...] without the use of any aid?”, with offered answers: no difficulty, some difficulty, a lot of difficulty, and cannot do at all/unable to do [28], bearing in mind fitting in the national context when completing the question, for example: “the length of five football fields” or “one city block” in […] [28].

The second category of walking difficulty (PF2) evaluated the ability to walk up or down 12 steps. For this purpose, the formulated question was, “Do you have difficulty walking up or down 12 steps?”, and offered answers included the following: no difficulty, some difficulty, a lot of difficulty, and cannot do at all/unable to do [28].

Four models of two physical functioning categories according to the degree of physical functioning impairment were proposed:

- Model 1: inability to perform PF1;
- Model 2: some/a lot of difficulty in performing PF1;
- Model 3: inability to perform PF2;
- Model 4: some/a lot of difficulty in performing PF2.

### 2.6. Statistical Analysis

Evaluated variables in this study are presented as whole numbers (n) and percentages (%). To assess the statistical significance between categorical variables, we performed the chi-squared test.

Univariate and multivariate (stepwise forward) logistic regression was performed for the identification of variables that are factors associated with PF1 and PF2 in four models with odds ratios (ORs) and 95% confidence intervals (CIs).

- Model 1: no difficulty in performing PF1 activity as referential value (0) versus unable to perform (1).
- Model 2: no difficulty in performing PF1 activity as referential value (0) versus some difficulty/a lot of difficulty (1).
- Model 3: no difficulty in performing PF2 activity as referential value (0) versus unable to perform (1).
- Model 4: no difficulty in performing PF2 activity as referential value (0) versus some difficulty/a lot of difficulty (1).

All variables that were found to be statistically significant in univariate logistic regression analysis were included in multivariate stepwise forward logistic regression analysis. All statistical analyses were performed using IBM SPSS statistics for Windows, version 22.0 (IBM Corp., Armonk, NY, USA).

The statistical significance was set at *p* < 0.05.

## 3. Results

Frequencies of sociodemographic characteristics in males regarding differences in performing the PF1 activity are presented in Table 1. Males with no difficulty in executing the PF1 activity were most frequently of age between 65 and 74 years (64.8%), while those that were unable to perform such activity were most frequently of age between 75 and 84 years (46.2%). Elderly males with no difficulty in performing PF1 were around 3.5 times more frequently married (77.7%), more frequently with a high school level of education (44.2%), and more frequently living in the city (56.0%), while those that were unable to perform such activity were less than twice more frequently married (67.9%), more frequently with an elementary level of education (50.0%), and more frequently from other areas of residency (57.5%). Obese elderly males were more than twice as frequently unable to perform the PF1 activity (33.3%) compared to those without difficulty (15.4%). Elderly males with a lower wealth index most frequently had a lot of difficulty in performing the PF1 activity (64.8%), while those with an upper wealth index were most frequently without difficulty in performing the PF1 activity (32.4%).

Frequencies of sociodemographic characteristics in males regarding differences in performing the PF2 activity are presented in Table 2. Elderly males with no difficulty in executing the PF2 activity were most frequently of age between 65 and 74 years (66.4%), while those that were unable to perform such activity were most frequently of age between 75 and 84 years (50.5%). Elderly males with no difficulty in performing PF2 were more than 3.5 times more frequently married (78.9%), more frequently in high school (43.1%), and more frequently living in the city (56.5%), while those that were unable to perform such activity were more than 2.5 times more frequently married (73.1%), more frequently with an elementary level of education (49.5%), and more frequently from other areas of residence (59.1%). Being unable to perform the PF2 activity was 1.5 times more frequent in obese elderly males (24.5%) compared to those without difficulty (14.8%). A lot of difficulty in performing the PF2 activity (68.1%) was most frequent in elderly males with a lower wealth index, while those without difficulty in performing the PF2 activity were most with an upper wealth index (33.7%).

Frequencies of sociodemographic characteristics in females regarding differences in performing the PF1 activity are presented in Table 3. Females with no difficulty in executing the PF1 activity were most frequently of age between 65 and 74 years (71.9%), while those that were unable to perform such activity were most frequently of age between 75 and 84 years (53.6%). Elderly females who were unable to perform the PF1 activity were less than twice as frequently single (65.3%). Elderly females were more frequently at the elementary school educational level in all categories of the PF1 activity. Obese elderly females were less than 1.5 times more frequently performing the PF1 activity with a lot of difficulty (32.1%) compared to those without difficulty (22.1%). Elderly females with a lower wealth index were most frequently with a lot of difficulty in performing the PF1 activity (65.2%), while those with an upper wealth index were most frequently without difficulty in performing the PF1 activity (34.3%). Those elderly females living in the city were more frequently without difficulty in performing the PF1 activity (63.2%), while those unable to perform the PF1 activity were more frequently from other areas of residence (56.0%).

Frequencies of sociodemographic characteristics in females regarding differences in performing the PF2 activity are presented in Table 4. Females with no difficulty in executing the PF2 activity were most frequently of age between 65 and 74 years (73.5%), while those that were unable to perform such activity were most frequently of age between 75 and 84 years (54.2%). Single elderly females who were unable to perform the PF2 activity were more than twice as frequent (67.9%). Elderly females were more frequently at the elementary school educational level in all categories of the PF2 activity. Obese elderly females were more than 1.5 times more frequently performing the PF2 activity with a lot of difficulty (33.1%) compared to those without difficulty (21.3%). Elderly females with a lower wealth index were most frequently with a lot of difficulty in performing the PF2 activity (66.7%), while those with an upper wealth index were most frequently without difficulty in performing the PF2 activity (36.0%). Those elderly females living in the city were more frequently without difficulty in performing the PF2 activity (62.5%), while those unable to perform the PF2 activity were more frequently from other areas of residence (55.8%).

Logistic regression analyses of the sociodemographic factors that were associated with different degrees of both tasks’ performances in elderly males are presented in Table 5. Statistically significant variables from the univariate logistic regression model were included in the multivariate stepwise forward logistic regression analysis, where age and educational levels were significantly associated with all models (Model 1–4), marital status with Model 4, and wealth index with Models 2, 3, and 4 in elderly males.

Logistic regression analyses of sociodemographic factors that were associated with different degrees of both tasks’ performances in elderly females are presented in Table 6. Statistically significant variables from the univariate logistic regression model were included in the multivariate stepwise forward logistic regression analysis, where age and educational levels were significantly associated with all models (Model 1–4), BMI and wealth index with Models 2 and 4, marital status with Model 2, and place of residence with Models 1 and 3 in elderly females.

## 4. Discussion

The findings from our study revealed that for elderly males, factors associated with being unable to perform the PF1 activity were increased age and a lower education level, while factors associated with some/a lot of difficulties in performing the PF1 activity were increased age, a lower education level, and wealth index. Regarding the inability to perform the PF2 activity in elderly males, factors associated with such functional impairment were increased age, a lower education level, and wealth index, while factors associated with some/a lot of difficulties in performing the PF2 activity were increased age, being single, a lower education level, and wealth index. For elderly females, factors associated with being unable to perform the PF1 activity were increased age, a lower education level, and living in another area of residence, while factors associated with some/a lot of difficulties in performing the PF1 activity were increased age and BMI, being single, a lower education level and wealth index. Regarding the inability to perform the PF2 activity in elderly females, factors associated with such functional impairment were increased age, a lower education level, and living in another area of residence, while factors associated with some/a lot of difficulties in performing the PF2 activity were increased age and BMI and a lower education level and wealth index.

Regarding the age of the study participants, the proportion of elderly increased more in the group aged 85 years and above than in the age group between 75 and 84 years, with a higher proportion in elderly females as the degree of difficulty in performing both the PF1 and PF2 activities increased, while the proportion of elderly age between 65 and 74 years decreased, with a higher proportion in elderly females. Regarding aging, it should be noticed that the range of reserve capacity or adaptability is reduced during aging as well as that the course of aging has much interindividual variability [8]. Previously, in a cross-sectional study performed on elderly people in a community setting, it was stated that multimorbidity is associated with an increased risk of functional limitation and an unfavorable quality of life [29]. Furthermore, it was stated that 62% of the elderly aged between 65 and 74 years and 81.5% of the ones 85 years and above had multimorbidities [30]. In an observational retrospective multicenter study, multimorbidity patterns, such as cardiometabolic, mechanical, and psychogeriatric patterns, in older populations were described as well, where it was pointed out that more females than males had at least one specific multimorbidity pattern as well as more than one multimorbidity pattern simultaneously [31]. Moreover, from a Vietnam Aging Survey from 2011, males were shown to have more chronic conditions associated with higher mortality rates, while females had chronic conditions that are non-life threatening in a nationally representative sample of individuals 60 years of age and above [32]. Additionally, in a multilevel study of older adults who participated in the Brazilian Health Survey from 2013, it was reported that higher gender differences in disability were associated with the highest rates of social gender inequality, where females were more affected [33]. All of this suggests the complex nature and influence of numerous factors in elderly males and females that affect functional capacity particularly. Therefore, it is important to propose and implement interventional measures on all levels of health care in order to prevent, timely recognize, and optimally treat potential conditions, as well as to improve functioning that should be adapted to the age and gender of the elderly individuals.

Our study demonstrated that married elderly males and single elderly females were more frequently with different degrees of physical disability. Single elderly females had the highest proportion of participants within the “unable to do” category for the PF2 activity compared to elderly males that were within the “a lot of difficulty” category for the same activity, while married elderly females had the lowest proportion of participants in “unable to do category” for the PF2 activity compared to elderly males that were within the “a lot of difficulty” category for the same activity. Previously, in a study with data based on the 2013 Disability and Use of Time (DUST) supplement to the 2013 Panel Study of Income Dynamics (PSID), it was reported that three-quarters of males and nearly half of females aged 65 and above are married or in a partnership [34]. This proportion is similar to our study, where elderly males were less than 3 times more frequently married, while elderly females were more than half of the study sample single. Bearing in mind that disability is a chronic stressor in older adults, present social support could reduce the emotional impact of such stressors [34]. From an empirical analysis of four surveys from the China Health and Retirement Longitudinal Study (CHARLS) that were performed in 2011, 2013, 2015, and 2018 years, individuals who were married were likely to have healthy lifestyles and more exercise, while elderly individuals who were widowed might have a lack of social connections which could lead to a decrease in health status [35]. Moreover, from a study on participants 60 years and older from nationally representative data of Bangladesh’s Household Income and Expenditure Survey (HIES)—2010, functional and self-care disabilities were proportionally higher in elderly females than in elderly males [36]. Considering the fact that numerous factors are interconnected when it comes to terms of marital status and disability in the elderly, along with their potential different degrees of impact on males and females, in proposing and implementing interventions in different healthcare settings, multidisciplinary, interdisciplinary, and transdisciplinary approaches are advised for optimal functional outcomes and improvement in health status.

Considering the education level of the study participants, the proportion of the elderly increased more in the group with an elementary education, while the proportion of elderly with university-level education decreased, with a higher proportion in elderly females and more for the PF2 activity in both genders. Education is considered a contributor to cognitive reserve development, where lower educational attainment can predict in later life poorer cognitive performance, and being at the primary school level or having none is associated with an increased risk of dementia (60%) [37]. In a nationally representative longitudinal study from China on community-dwelling individuals 60 years of age and above, educational attainments were shown to have positive associations with disability-free life expectancy as well as total life expectancy, particularly more in females [38]. Furthermore, in a population-based cross-sectional study from Brazil that was conducted in 2008, it was noticed that low educational levels were shown to be associated with disabilities in basic and instrumental activities in the elderly [39]. Moreover, our findings stressed that elderly females with a university-level education had a higher proportional decrease than elderly males as the degree of difficulty in physical functioning limitations increased. Therefore, affirmation and promotion of education and implementation of measures for inclusion in higher educational activities are important, particularly in early life.

Regarding BMI, our results demonstrated that there was a higher increase in the proportion of elderly who were underweight than obese for both physical functioning activities, while overweight decreased in proportion. More increases in the proportion of underweight were noticed in elderly males than females. In older adults, obesity was shown to be associated with cardiovascular and metabolic diseases and cancers as well as with an increase in disability and functional limitations [40]. In a longitudinal observational study with data from the International Mobility in Aging Study (IMIAS), older adults with abdominal obesity were reported to have higher mobility disability [41]. Moreover, in a community-based cross-sectional analytical study on older adults 60 years of age and above, it was reported that among underweight elderly, functional impairments were more prevalent [42]. Our findings pointed out that being overweight in the elderly is, to a certain degree, more favorable in terms of physical functioning impairment compared to being underweight or obese.

For the wealth index changes, the proportion of elderly with a lower wealth index increased with an increase in the difficulty degree of both types of physical functioning activities, while the proportion of those with an upper wealth index level decreased with an increase in the difficulty degree of both types of physical functioning activities. Previously, it was stated that older adults with the highest wealth, education, and social class levels were shown to have the lowest disability rates, with a stronger association of socioeconomic position with disability for males [43]. Moreover, in another study, elderly females with a higher wealth index had significantly lower odds of functional disability compared to females with a lower wealth index, while the odds of functional disability for elderly males did not significantly differ between lower and higher wealth index groups [36]. The findings from the cross-sectional study on older adults from the International Mobility in Aging Study pointed out that males report greater community disability and that occupation and education are shown to be factors associated with a decrease in participation frequency for males, while females report greater perceived disability and occupation and income as factors associated with a decrease in participation frequency [9].

### 4.1. Study Limitations

This cross-sectional design study does not support causal analysis but can provide insights into significant associations and factors whose predictive roles can be explored. This study does not include elderly individuals living in community organizations, thereby highlighting the need for specific research to address their needs. Furthermore, this study advocates for increased community engagement by promoting community-building initiatives that involve elderly individuals facing economic challenges. However, the set of study variables does not provide economic estimates, and future research should be conducted to address this gap.

### 4.2. Policy and Practice Implications

The findings from this study underscore critical aspects of functional performance among elderly males and females, which policymakers should consider when developing targeted intervention programs aimed at enhancing physical function through tailored exercise regimens, educational resources, and support services. In terms of exercise programs, practitioners are encouraged to design physical activity initiatives that are customizable to individual needs and relevant to their experiences, emphasizing skill-building and safe practices—particularly for individuals with lower educational and economic resources. Given that the wealth index serves as a predictor of challenges in physical functioning, policies aimed at improving access to resources for low-income elderly individuals may include subsidization of fitness programs, the establishment of accessible community centers, and the provision of social services to assist with daily living activities. Furthermore, support services should incorporate training programs for caregivers that highlight the significance of educational and socioeconomic factors influencing the elderly’s capacity to engage in physical activities, equipping them with strategies to assist individuals facing these challenges. A multidisciplinary approach is advocated, involving collaboration among physical therapists, occupational therapists, social workers, caregivers, and educators to effectively support elderly individuals in overcoming barriers related to physical functioning activities.

## 5. Conclusions

Increased age and a lower educational level were factors for both types of physical activity impairments in elderly males and females. Being single was a factor associated with impairment to perform ascending and descending 12 steps in elderly males. In contrast, in elderly females, it was a factor associated with impairment to perform walking on ground level half a km without the use of any aid. A lower wealth index was associated with physical activity impairments in elderly males and females. Residing in a place other than the city was associated with both types of physical activity impairments in elderly females.

Our study findings point to the importance of adequate and optimal creation and implementation of gender-specific social strategies and interventions that should be oriented towards increasing awareness and promotion of social support as well as improving the participation, mobility, and functioning of the elderly. The effectiveness of such strategies and interventions, as well as their implementation, should be monitored and evaluated in future research.

## Figures and Tables

**Table 1:** Frequencies of sociodemographic characteristics in males regarding differences in performing PF1 activity.

Self-Perceived General Health n (%)	No Difficulty	Some Difficulty	A Lot of Difficulty	Unable To Do	Total	*p*
PF1 Activity						
Age						
65–74 years	581 (64.8)	151 (51.0)	100 (43.5)	43 (40.6)	875 (57.3)	<0.01
75–84 years	295 (32.9)	133 (44.9)	112 (48.7)	49 (46.2)	589 (38.5)	<0.01
≥85 years	20 (2.2)	12 (4.1)	18 (7.8)	14 (13.2)	64 (4.2)	<0.01
*p*	<0.001	<0.001	<0.001	<0.001	<0.001	
Marital status						
Single	200 (22.3)	81 (27.4)	80 (34.8)	34 (32.1)	395 (25.9)	<0.05
Married	696 (77.7)	215 (72.6)	150 (65.2)	72 (67.9)	1133 (74.1)	<0.05
*p*	<0.001	<0.001	<0.01	<0.01	<0.001	
Education level						
Elementary	294 (32.8)	137 (46.3)	104 (45.2)	53 (50.0)	588 (38.5)	<0.05
High school	396 (44.2)	102 (34.5)	94 (40.9)	39 (36.8)	631 (41.3)	<0.05
University	206 (23.0)	57 (19.3)	32 (13.9)	14 (13.2)	309 (20.2)	<0.05
*p*	<0.05	<0.05	<0.01	<0.01	<0.05	
BMI						
Underweight	3 (0.4)	7 (2.8)	6 (3.4)	3 (5.0)	19 (1.5)	<0.05
Normal weight	302 (38.7)	86 (34.8)	62 (35.0)	22 (36.7)	472 (37.3)	>0.05
Overweight	356 (45.6)	112 (45.3)	73 (41.2)	15 (25.0)	556 (44.0)	<0.05
Obese	120 (15.4)	42 (17.0)	36 (20.3)	20 (33.3)	218 (17.2)	<0.05
*p*	<0.001	<0.001	<0.001	<0.001	<0.001	
Wealth Index						
Lower	443 (49.4)	175 (59.1)	149 (64.8)	67 (63.2)	834 (54.6)	<0.01
Middle	163 (18.2)	43 (14.5)	43 (18.7)	19 (17.9)	268 (17.5)	>0.05
Upper	290 (32.4)	78 (26.4)	38 (16.5)	20 (18.9)	426 (27.9)	<0.05
*p*	<0.01	<0.001	<0.001	<0.001	<0.001	
Place of residence						
City	502 (56.0)	160 (54.1)	95 (41.3)	45 (42.5)	802 (52.5)	<0.05
Another place	394 (44.0)	136 (45.9)	135 (58.7)	61 (57.5)	726 (47.5)	<0.05
*p*	<0.05	<0.05	<0.05	<0.05	<0.05	

PF—physical functioning; BMI—body mass index; *p*—chi-squared test.

**Table 2:** Frequencies of sociodemographic characteristics in males regarding differences in performing PF2 activity.

Self-Perceived General Health n (%)	No Difficulty	Some Difficulty	A Lot of Difficulty	Unable To Do	Total	*p*
PF2 Activity						
Age						
65–74 years	542 (66.4)	176 (50.9)	119 (43.6)	38 (40.9)	875 (57.3)	<0.001
75–84 years	256 (31.4)	155 (44.8)	131 (48.0)	47 (50.5)	589 (38.5)	<0.01
≥85 years	18 (2.2)	15 (4.3)	23 (8.4)	8 (8.6)	64 (4.2)	<0.05
*p*	<0.001	<0.001	<0.001	<0.001	<0.001	
Marital status						
Single	172 (21.1)	104 (30.1)	94 (34.4)	25 (26.9)	395 (25.9)	<0.05
Married	644 (78.9)	242 (69.9)	179 (65.6)	68 (73.1)	1133 (74.1)	<0.01
*p*	<0.001	<0.001	<0.001	<0.001	<0.001	
Education level						
Elementary	262 (32.1)	143 (41.3)	137 (50.2)	46 (49.5)	588 (38.5)	<0.05
High school	352 (43.1)	138 (39.9)	104 (38.1)	37 (39.8)	631 (41.3)	> 0.05
University	202 (24.8)	65 (18.8)	32 (11.7)	10 (10.8)	309 (20.2)	<0.01
*p*	<0.05	<0.05	<0.01	<0.01	<0.05	
BMI						
Underweight	3 (0.4)	8 (2.8)	4 (2.0)	4 (7.5)	19 (1.5)	<0.01
Normal weight	288 (40.2)	91 (31.4)	73 (35.6)	20 (37.7)	472 (37.3)	<0.05
Overweight	320 (44.6)	141 (48.6)	79 (38.5)	16 (30.2)	556 (44.0)	<0.05
Obese	106 (14.8)	50 (17.2)	49 (23.9)	13 (24.5)	218 (17.2)	<0.05
*p*	<0.001	<0.001	<0.001	<0.001	<0.001	
Wealth index						
Lower	386 (47.3)	202 (58.4)	186 (68.1)	60 (64.5)	834 (54.6)	<0.05
Middle	155 (19.0)	53 (15.3)	41(15.0)	19 (20.4)	268 (17.5)	>0.05
Upper	275 (33.7)	91 (26.3)	46 (16.8)	14 (15.1)	426 (27.9)	<0.05
*p*	<0.01	<0.01	<0.001	<0.001	<0.01	
Place of residence						
City	461 (56.5)	191 (55.2)	112 (41.0)	38 (40.9)	802 (52.5)	<0.01
Another place	355 (43.5)	155 (44.8)	161 (59.0)	55 (59.1)	726 (47.5)	<0.01
*p*	<0.05	<0.05	<0.01	<0.01	<0.05	

PF—physical functioning; BMI—body mass index; *p*—chi-squared test.

**Table 3:** Frequencies of sociodemographic characteristics in females regarding differences in performing PF1 activity.

Self-Perceived General Health n (%)	No Difficulty	Some Difficulty	A Lot of Difficulty	Unable To Do	Total	*p*
PF1 Activity						
Age						
65–74 years	537 (71.9)	280 (51.3)	181 (42.3)	82 (28.2)	1080 (53.7)	<0.001
75–84 years	195 (26.1)	237 (43.4)	208 (48.6)	156 (53.6)	796 (39.6)	<0.01
≥85 years	15 (2.0)	29 (5.3)	39 (9.1)	53 (18.2)	136 (6.8)	<0.001
*p*	<0.001	<0.001	<0.001	<0.001	<0.001	
Marital status						
Single	371 (49.7)	338 (61.9)	281 (65.7)	190 (65.3)	1180 (58.6)	<0.01
Married	376 (50.3)	208 (38.1)	147 (34.3)	101 (34.7)	832 (41.4)	<0.05
*p*	>0.05	<0.01	<0.01	<0.01	<0.05	
Education level						
Elementary	395 (52.9)	401 (73.4)	337 (78.7)	235 (80.8)	1368 (68.0)	<0.001
High school	246 (32.9)	114 (20.9)	71 (16.6)	42 (14.4)	473 (23.5)	<0.01
University	106 (14.2)	31 (5.7)	20 (4.7)	14 (4.8)	171 (8.5)	<0.05
*p*	<0.01	<0.001	<0.001	<0.001	<0.001	
BMI						
Underweight	16 (2.8)	10 (2.9)	5 (2.2)	7 (5.4)	38 (3.0)	<0.05
Normal weight	192 (34.2)	102 (29.7)	62 (27.7)	48 (37.2)	404 (32.1)	<0.05
Overweight	230 (40.9)	140 (40.8)	85 (37.9)	41 (31.8)	496 (39.4)	<0.05
Obese	124 (22.1)	91 (26.5)	72 (32.1)	33 (25.6)	320 (25.4)	<0.05
*p*	<0.001	<0.001	<0.001	<0.001	<0.001	
Wealth Index						
Lower	339 (45.4)	340 (62.3)	279 (65.2)	183 (62.9)	1141 (56.7)	<0.01
Middle	152 (20.3)	95 (17.4)	67 (15.7)	49 (16.8)	363 (18.0)	<0.05
Upper	256 (34.3)	111 (20.3)	82 (19.2)	59 (20.3)	508 (25.2)	<0.05
*p*	<0.05	<0.001	<0.001	<0.001	<0.001	
Place of residence						
City	472 (63.2)	299 (54.8)	202 (47.2)	128 (44.0)	1101 (54.7)	<0.01
Another place	275 (36.8)	247 (45.2)	226 (52.8)	163 (56.0)	911 (45.3)	<0.01
*p*	<0.01	<0.05	<0.05	<0.05	<0.05	

PF—physical functioning; BMI—body mass index; *p*—chi-squared test.

**Table 4:** Frequencies of sociodemographic characteristics in females regarding differences in performing PF2 activity.

Self-Perceived General Health. n (%)	No Difficulty	Some Difficulty	A Lot of Difficulty	Unable To Do	Total	*p*
PF2 Activity						
Age						
65–74 years	433 (73.5)	335 (55.0)	243 (43.0)	69 (27.7)	1080 (53.7)	<0.001
75–84 years	146 (24.9)	238 (39.1)	277 (49.0)	135 (54.2)	796 (39.6)	<0.01
≥85 years	10 (1.7)	36 (5.9)	45 (8.0)	45 (18.1)	136 (6.8)	<0.05
*p*	<0.001	<0.001	<0.001	<0.001	<0.001	
Marital status						
Single	289 (49.1)	356 (58.5)	366 (64.8)	169 (67.9)	1180 (58.6)	<0.05
Married	300 (50.9)	253 (41.5)	199 (35.2)	80 (32.1)	832 (41.4)	<0.01
*p*	>0.05	<0.05	<0.05	<0.05	<0.05	
Education level						
Elementary	304 (51.6)	416 (68.3)	442 (78.2)	206 (82.7)	1368 (68.0)	<0.001
High school	184 (31.2)	158 (25.9)	99 (17.5)	32 (12.9)	473 (23.5)	<0.001
University	101 (17.1)	35 (5.7)	24 (4.2)	11 (4.4)	171 (8.5)	<0.01
*p*	<0.01	<0.001	<0.001	<0.001	<0.001	
BMI						
Underweight	10 (2.2)	11 (2.8)	7 (2.3)	10 (8.9)	38 (3.0)	<0.05
Normal weight	158 (35.3)	119 (30.7)	84 (27.0)	43 (38.4)	404 (32.1)	<0.05
Overweight	184 (41.2)	164 (42.3)	117 (37.6)	31 (27.7)	496 (39.4)	<0.05
Obese	95 (21.3)	94 (24.2)	103 (33.1)	28 (25.0)	320 (25.4)	<0.05
*p*	<0.001	<0.001	<0.001	<0.001	<0.001	
Wealth index						
Lower	261 (44.3)	343 (56.3)	377 (66.7)	160 (64.3)	1141 (56.7)	<0.01
Middle	116 (19.7)	118 (19.4)	86 (15.2)	43 (17.3)	363 (18.0)	>0.05
Upper	212 (36.0)	148 (24.3)	102 (18.1)	46 (18.5)	508 (25.2)	<0.05
*p*	<0.01	<0.01	<0.001	<0.001	<0.01	
Place of residence						
City	368 (62.5)	354 (58.1)	269 (47.6)	110 (44.2)	1101 (54.7)	<0.01
Another place	221 (37.5)	255 (41.9)	296 (52.4)	139 (55.8)	911 (45.3)	<0.01
*p*	<0.01	<0.05	<0.05	<0.05	<0.05	

PF—physical functioning; BMI—body mass index; *p*—chi-squared test.

**Table 5:** Sociodemographic factors associated with executing PF1 and PF2 activities in males.

Variables	Model 1 OR (95% CI)	Model 2 OR (95% CI)	Model 3 OR (95% CI)	Model 4 OR (95% CI)
Univariate logistic regression				
Age	2.667 *** (1.921–3.702)	1.902 *** (1.569–2.305)	2.530 *** (1.774–3.609)	2.024 *** (1.676–2.444)
BMI	1.227 (0.859–1.753)	1.027 (0.874–1.207)	0.965 (0.656–1.421)	1.165 (0.997–1.362)
Marital status	0.612 * (0.395–0.947)	0.651 ** (0.511–0.831)	0.731 (0.448–1.191)	0.568 *** (0.447–0.721)
Education level	0.595 *** (0.444–0.796)	0.704 *** (0.607–0.817)	0.550 *** (0.403–0.751)	0.666 *** (0.577–0.769)
Wealth index	0.686 ** (0.535–0.881)	0.738 *** (0.650–0.839)	0.601 *** (0.456–0.792)	0.696 *** (0.616–0.788)
Place of residence	1.731 ** (1.152–2.600)	1.354 ** (1.091–1.680)	1.884 ** (1.218–2.913)	1.354 ** (1.098–1.670)
Multivariate (stepwise forward) logistic regression				
Age	2.591 *** (1.861–3.607)	1.791 *** (1.472–2.180)	2.386 *** (1.664–3.422)	1.883 *** (1.552–2.285)
BMI	-	-	-	-
Marital status	-	-	-	0.688 ** (0.536–0.882)
Education level	0.626 ** (0.469–0.836)	0.811 * (0.685–0.960)	0.697 * (0.491–0.990)	0.784 ** (0.665–0.924)
Wealth index	-	0.823 ** (0.712–0.952)	0.724 * (0.530–0.991)	0.787 ** (0.683–0.906)
Place of residence	-	-	-	-

BMI—body mass index; OR—odds ratio; CI—confidence interval; * *p* < 0.05; ** *p* < 0.01; *** *p* < 0.001.

**Table 6:** Sociodemographic factors associated with difficulties in executing PF1 and PF2 activities in females.

Variables	Model 1 OR (95% CI)	Model 2 OR (95% CI)	Model 3 OR (95% CI)	Model 4 OR (95% CI)
Univariate logistic regression				
Age	5.020 *** (3.906–6.451)	2.541 *** (2.119–3.047)	5.577 *** (4.199–7.406)	2.592 *** (2.133–3.150)
BMI	0.933 (0.738–1.178)	1.204 * (1.043–1.391)	0.829 (0.643–1.068)	1.212 * (1.046–1.404)
Marital status	0.525 *** (0.396–0.695)	0.566 *** (0.466–0.687)	0.456 *** (0.334–0.622)	0.603 *** (0.494–0.736)
Education level	0.379 *** (0.294–0.490)	0.466 *** (0.399–0.544)	0.332 *** (0.250–0.442)	0.468 *** (0.402–0.544)
Wealth index	0.648 *** (0.549–0.764)	0.638 *** (0.570–0.715)	0.596 *** (0.496–0.715)	0.653 *** (0.582–0.732)
Place of residence	2.186 *** (1.660–2.878)	1.620 *** (1.334–1.969)	2.104 *** (1.558–2.842)	1.473 *** (1.203–1.803)
Multivariate (stepwise forward) logistic regression				
Age	4.708 *** (3.634–6.100)	2.354 *** (1.839–3.012)	4.985 *** (3.714–6.691)	2.772 *** (2.150–3.574)
BMI	-	1.348 *** (1.150–1.579)	-	1.329 ** (1.131–1.563)
Marital status	-	0.713 * (0.550–0.925)	-	-
Education level	0.537 *** (0.406–0.711)	0.653 *** (0.535–0.798)	0.494 *** (0.363–0.672)	0.639 *** (0.526–0.777)
Wealth index	-	0.740 *** (0.633–0.864)	-	0.731 *** (0.625–0.855)
Place of residence	1.704 *** (1.226–2.367)	-	1.575 * (1.095–2.264)	-

BMI—body mass index; OR—odds ratio; CI—confidence interval; * *p* < 0.05; ** *p* < 0.01; *** *p* < 0.001.

## Data Availability

Data are available upon reasonable request from the first author.

## References

[1] National Institute of Aging Global Aging. https://www.nia.nih.gov/research/dbsr/global-aging.

[2] Takele M.D., Eriku G.A., Merawie D.M., Zinabu F.S., Fentanew M., Belay G.J., Kibret A.K. (2024). Functional disability and its associated factors among community-dweller older adults living in Gondar Town, Ethiopia: A community-based cross-sectional study. BMC Public Health.

[3] World Health Organization Ageing: Global Population. https://www.who.int/news-room/questions-and-answers/item/population-ageing.

[4] World Health Organization Aging and Health. https://www.who.int/news-room/fact-sheets/detail/ageing-and-health.

[5] United Nations (2024). GLOBAL ISSUES Ageing. https://www.un.org/zh/global-issues/ageing.

[6] Sevo G., Davidovic M., Erceg P., Despotovic N., Milosevic D.P., Tasic M. (2015). On aging and aged care in Serbia. J. Cross Cult. Gerontol..

[7] Statistical Office of the Republic of Serbia (2022). Population—Population Estimations. Estimated Population. https://www.stat.gov.rs/en-us/vesti/statisticalrelease/?p=13622.

[8] Molton I.R., Jensen M.P. (2010). Aging and disability: Biopsychosocial perspectives. Phys. Med. Rehabil. Clin. N. Am..

[9] Corrêa L.C.A.C., Gomes C.D.S., Camara S.M.A.D., Barbosa J.F.S., Azevedo I.G., Vafaei A., Guerra R.O. (2023). Gender-Specific Associations between Late-Life Disability and Socioeconomic Status: Findings from the International Mobility and Aging Study (IMIAS). Int. J. Environ. Res. Public Health.

[10] Kostadinovic M., Nikolic D., Petronic I., Cirovic D., Grajic M., Santric Milicevic M. (2018). Sociodemographic Predictors of Physical Functioning in the Elderly: A National Health Survey. Int. J. Environ. Res. Public Health.

[11] Chad K.E., Reeder B.A., Harrison E.L., Ashworth N.L., Sheppard S.M., Schultz S.L., Bruner B.G., Fisher K.L., Lawson J.A. (2005). Profile of physical activity levels in community-dwelling older adults. Med. Sci. Sports Exerc..

[12] Tsimbos C., Verropoulou G. (2008). A multivariate analysis of factors associated with physical functioning limitations in the Greek elderly population. J. Stat. Manag. Syst..

[13] Liu H., Wang M. (2022). Socioeconomic status and ADL disability of the older adults: Cumulative health effects, social outcomes and impact mechanisms. PLoS ONE.

[14] Zhong Y., Wang J., Nicholas S. (2017). Gender, childhood and adult socioeconomic inequalities in functional disability among Chinese older adults. Int. J. Equity Health.

[15] Abellán A., Rodríguez-Laso Á., Pujol R., Barrios L. (2015). A higher level of education amplifies the inverse association between income and disability in the Spanish elderly. Aging Clin. Exp. Res..

[16] Fong J.H. (2019). Disability incidence and functional decline among older adults with major chronic diseases. BMC Geriatr..

[17] Finlayson M.L., Peterson E.W. (2010). Falls, aging, and disability. Phys. Med. Rehabil. Clin. N. Am..

[18] Matsuda P.N., Verrall A.M., Finlayson M.L., Molton I.R., Jensen M.P. (2015). Falls among adults aging with disability. Arch. Phys. Med. Rehabil..

[19] Ma T., Lu D., Zhu Y.S., Chu X.F., Wang Y., Shi G.P., Wang Z.D., Yu L., Jiang X.Y., Wang X.F. (2018). ACTN3 genotype and physical function and frailty in an elderly Chinese population: The Rugao Longevity and Ageing Study. Age Ageing.

[20] Delmonico M.J., Kostek M.C., Doldo N.A., Hand B.D., Walsh S., Conway J.M., Carignan C.R., Roth S.M., Hurley B.F. (2007). Alpha-actinin-3 (ACTN3) R577X polymorphism influences knee extensor peak power response to strength training in older men and women. J. Gerontol. A Biol. Sci. Med. Sci..

[21] Kikuchi N., Yoshida S., Min S., Lee K., Sakamaki-Sunaga M., Okamoto T., Nakazato K. (2015). The ACTN3 R577X genotype is associated with muscle function in a Japanese population. Appl. Physiol. Nutr. Metab..

[22] Results of Health Research in Serbia. http://www.batut.org.rs/download/publikacije/IstrazivanjeZdravljaStanovnistvaRS2013.pdf.

[23] Radosavljevic N., Nikolic D., Lazovic M., Petronic I., Milicevic V., Radosavljevic Z., Potic J., Ilic-Stojanovic O., Jeremic A. (2013). Estimation of functional recovery in patients after hip fracture by Berg Balance Scale regarding the sex, age and comorbidity of participants. Geriatr. Gerontol. Int..

[24] World Health Organization (2000). Obesity: Preventing and Managing the Global Epidemic.

[25] Jankovic J., Simic S., Marinkovic J. (2010). Inequalities that hurt: Demographic, socio-economic and health status inequalities in the utilization of health services in Serbia. Eur. J. Public Health.

[26] Rutstein S.O., Johnson K. (2004). The DHS Wealth Index. DHS Comparative Reports No. 6.

[27] Gudelj Rakic J.M. (2016). Relationship between Obesity and Health Behaviours in the Serbian Adult Population. Ph.D. Thesis.

[28] European Health Interview Survey (EHIS Wave 2) Methodological Manual. http://ec.europa.eu/eurostat/documents/3859598/5926729/KS-RA-13-018-EN.PDF/26c7ea80-01d8-420e-bdc6-e9d5f6578e7c.

[29] Sembiah S., Dasgupta A., Taklikar C.S., Paul B., Bandyopadhyay L., Burman J., Pawar N., Subbakrishna N. (2022). Gender inequalities in prevalence, pattern and predictors of multimorbidity among geriatric population in rural West Bengal. J. Family Med. Prim. Care.

[30] Salive M.E. (2013). Multimorbidity in older adults. Epidemiol. Rev..

[31] Abad-Díez J.M., Calderón-Larrañaga A., Poncel-Falcó A., Poblador-Plou B., Calderón-Meza J.M., Sicras-Mainar A., Clerencia-Sierra M., Prados-Torres A. (2014). Age and gender differences in the prevalence and patterns of multimorbidity in the older population. BMC Geriatr..

[32] Le D.D., Leon-Gonzalez R., Giang L.T. (2020). Decomposing gender inequality in functional disability among older people in Vietnam. Arch. Gerontol. Geriatr..

[33] Lima A.L.B., Espelt A., Bosque-Prous M., Lima K.C. (2020). Gender differences in disability among older adults in the context of social gender and income inequalities: 2013 Brazilian Health Survey. Rev. Bras. Epidemiol..

[34] Carr D., Cornman J.C., Freedman V.A. (2019). Do Family Relationships Buffer the Impact of Disability on Older Adults’ Daily Mood? An Exploration of Gender and Marital Status Differences. J. Marriage Fam..

[35] Pang J., Xu S., Wu Y. (2023). Effect of widowhood on the risk of disability among the elderly in China. Front. Psychiatry.

[36] Tareque M.I., Tiedt A.D., Islam T.M., Begum S., Saito Y. (2017). Gender differences in functional disability and self-care among seniors in Bangladesh. BMC Geriatr..

[37] Angrisani M., Lee J., Meijer E. (2020). The gender gap in education and late-life cognition: Evidence from multiple countries and birth cohorts. J. Econ. Ageing.

[38] Chen H., Li M., Zhang Y. (2025). Educational attainment and male-female health-survival paradox among older adults in China: A nationally representative longitudinal study. BMC Geriatr..

[39] Nunes J.D., Saes M.O., Nunes B.P., Siqueira F.C.V., Soares D.C., Fassa M.E.G., Thumé E., Facchini L.A. (2017). Functional disability indicators and associated factors in the elderly: A population-based study in Bagé, Rio Grande do Sul, Brazil. Epidemiol. Serv. Saude.

[40] Samper-Ternent R., Al Snih S. (2012). Obesity in Older Adults: Epidemiology and Implications for Disability and Disease. Rev. Clin. Gerontol..

[41] Fernandes de Souza Barbosa J., Dos Santos Gomes C., Vilton Costa J., Ahmed T., Zunzunegui M.V., Curcio C.L., Gomez F., Oliveira Guerra R. (2018). Abdominal Obesity and Mobility Disability in Older Adults: A 4-Year Follow-Up the International Mobility in Aging Study. J. Nutr. Health Aging.

[42] Ahamed F., Rehman T., Krishnamoorthy Y., Kaur A., Debnath A., Ghosh T. (2022). Underweight is an important predictor for functional impairment among the older adults in Urban West Bengal, India: A cross sectional analytical study. J. Family Med. Prim Care.

[43] Gjonça E., Tabassum F., Breeze E. (2009). Socioeconomic differences in physical disability at older age. J. Epidemiol. Community Health.

